# Comparing Two Distal Radial Hemostatic Devices for Radial Artery Patency Post-TACE: A Randomized Trial

**DOI:** 10.1007/s00270-025-04337-8

**Published:** 2026-01-23

**Authors:** Cheng-Chun Lee, Jia-Min Wu, Fen-Ni Tsai, Pao-Chia Chiu, Yu-Chen Chen, Ya-Lan Liang, Jen-I. Hwang, Kuan-Chun Hsueh

**Affiliations:** 1 Division of Diagnostic Radiology, Department of Medical Imaging, Tungs’ Taichung Metroharbor Hospital, No. 699, Section 8, Taiwan Boulevard, Wuqi District, Taichung City, 435 Taiwan (R.O.C.); 2 Master in Senior Health and Exercise Science, Tunghai University, No. 1727, Sec. 4, Taiwan Boulevard, Xitun District, Taichung City, 407 Taiwan (R.O.C.); 3 Bachelor’s Degree Program in Senior Wellness and Sports Science, Tunghai University, Taichung City, Taiwan (R.O.C.); 4 Department of Medical Research, Tungs’ Taichung Metroharbor Hospital, Taichung City, Taiwan (R.O.C.); 5 Division of General Surgery, Department of Surgery, Tungs’ Taichung Metroharbor Hospital, No. 699, Section 8, Taiwan Boulevard, Wuqi District, Taichung City, 435403 Taiwan (R.O.C.); 6 Department of Post-Baccalaureate Medicine, College of Medicine, National Chung Hsing University, No. 145, Xingda Rd., South Dist., Taichung City, 402 Taiwan (R.O.C.)

**Keywords:** Distal radial artery, Distal transradial access, Transarterial chemoembolization, Hepatocellular carcinoma, Vascular patency

## Abstract

**Purpose:**

To compare the patency outcomes and safety profile between the modified TR Band and the PreludeSYNC DISTAL (PSD) for hemostasis following distal transradial access (dTRA) for transarterial chemoembolization (TACE).

**Methods:**

This prospective randomized trial enrolled 104 participants undergoing TACE via dTRA (143 procedures, all performed using a 4-Fr catheter. Participants achieved hemostasis with either the modified TR Band (n = 74) or PSD (n = 69). The primary endpoint was the incidence of radial artery occlusion (RAO) assessed by Doppler ultrasonography at 4 h, 24 h, and > 1 week. Outcome assessors were blinded. Secondary endpoints included hemostatic performance and complications.

**Results:**

No complete RAO occurred in either group (0/143). Partial RAO (mural thrombus with preserved flow) peaked at 4 h (TR Band: 28.4% vs. PSD: 18.8%; risk difference 9.5%, 95% CI -4.3 to 23.3%, *p* = 0.181) and declined to 4.1% vs. 2.9% by final follow-up (*p* = 1.000), representing a spontaneous resolution rate of 85.3%. Generalized estimating equations showed no significant difference in overall incidence. The TR Band group had a numerically higher rate of delayed hemostasis (> 4 h) compared with PSD (5.4% vs. 0.0%, *p* = 0.121). Complication rates (hematoma, pseudoaneurysm) did not differ significantly.

**Conclusion:**

The modified TR Band and PSD demonstrated comparable rates of partial radial artery occlusion and access-site complications following dTRA for TACE.

**Graphical Abstract:**

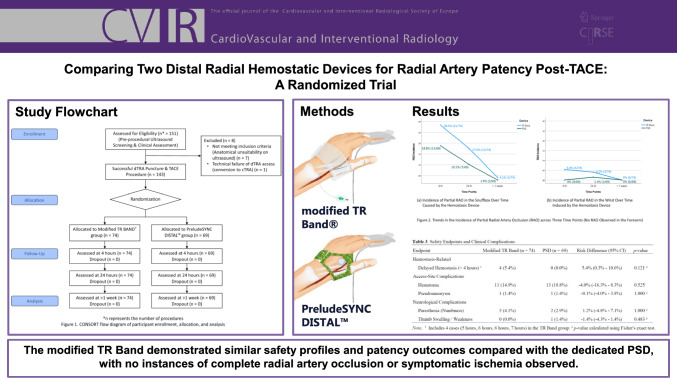

**Supplementary Information:**

The online version contains supplementary material available at 10.1007/s00270-025-04337-8.

## Introduction

Transarterial chemoembolization (TACE) is a standard-of-care therapy for patients with intermediate-stage hepatocellular carcinoma (HCC) [1]. While traditionally performed via the transfemoral route [2], transradial access (TRA) has emerged as an alternative approach for TACE, which is now commonly used in coronary procedures. TRA offers fewer access-site complications, improved patient comfort, shorter recovery times, and similar procedural outcome in selected patients [3–6].

There are two types of TRA: conventional transradial access (cTRA) at the wrist [7], and distal transradial access (dTRA), which targets the radial artery at the anatomical snuffbox [8]. Although the distal radial artery is smaller (mean diameters ranging from 1.71 mm in Asian populations to 2.40 mm in Western populations) [9], it remains accessible due to its superficial course and palpable pulse [10].

Three advantages drive the adoption of dTRA over conventional access. First, dTRA preserves the proximal radial trunk by puncturing distally, maintaining access for repeat interventions [11]. Second, dTRA offers ergonomic benefits for interventional radiologists. Left-sided dTRA allows the patient's arm to rest naturally across the abdomen, simulating conventional femoral access and optimizing operator workflow [12]. Third, the ANGIE trial demonstrated that dTRA yields a significantly lower rate of radial artery occlusion (RAO) (3.7% vs. 7.9%) and shorter hemostasis times (60 vs. 120 min) compared with conventional access [13].

RAO remains a recognized complication of TRA and may compromise future access in patients requiring repeat TACE [14]. While RAO incidence with cTRA varies widely in cardiovascular interventions [15], ultrasound-based studies in interventional radiology report occlusion rates as low as 0% following dTRA [16, 17]. Even with repeated dTRA, asymptomatic occlusion rates remain low at 4.1% [11]. Furthermore, dTRA may preserve the superficial palmar branch and collateral flow, reducing ischemic risk if occlusion occurs [18]. However, data on optimal hemostasis strategies following dTRA in TACE remain limited.

Hemostasis after dTRA can be achieved through manual compression [19] or the use of hemostasis devices, such as the TR Band® (Terumo Medical, Somerset, NJ, USA), designed initially for cTRA [20], or the PreludeSYNC DISTAL (PSD; Merit Medical Systems, South Jordan, UT, USA). The PSD is purpose-built for the distal radial artery, featuring a framed design that conforms to the snuffbox [21]. However, international consensus defines dTRA puncture sites to include both the anatomical snuffbox and the dorsum of the hand [22]. The rigid frame of the PSD may lack adaptability for these variable puncture sites, whereas the TR Band, when modified by removing the plastic support plate, offers flexible circumferential compression [20]. Whether the modified TR Band yields patency outcomes comparable to the PSD remains unknown.

Given the need for repeat access in HCC management [11, 23, 24], this prospective, randomized study compares the patency outcomes and safety profile of the modified TR Band versus the PSD following dTRA in TACE. The primary endpoint was the incidence of RAO assessed by Doppler ultrasonography [25]. Secondary endpoints included hemostatic performance and complications, hemodynamic parameters, and the feasibility of repeat access.

## Materials and Methods

### Research Participants

This study was conducted in accordance with the Declaration of Helsinki and approved by the Institutional Review Board (approval numbers: 111R56 and 113R41). From June 2022 to December 2024, all patients scheduled for TACE via dTRA were prospectively enrolled after obtaining written informed consent.

Inclusion criteria were (1) age ≥ 20 years, (2) clinical indication for TACE, and (3) a patent distal radial artery deemed suitable for dTRA, defined by the following ultrasound criteria: (a) clearly visualized vessel lumen, (b) vessel diameter ≥ 1.2 mm, and (c) absence of severe calcification or tortuosity, with a straight segment > 1 cm for puncture. Exclusion criteria were (1) existing RAO and (2) severe allergy or intolerance to contrast agents or related medications.

### Research Design

**Fig. 1:**
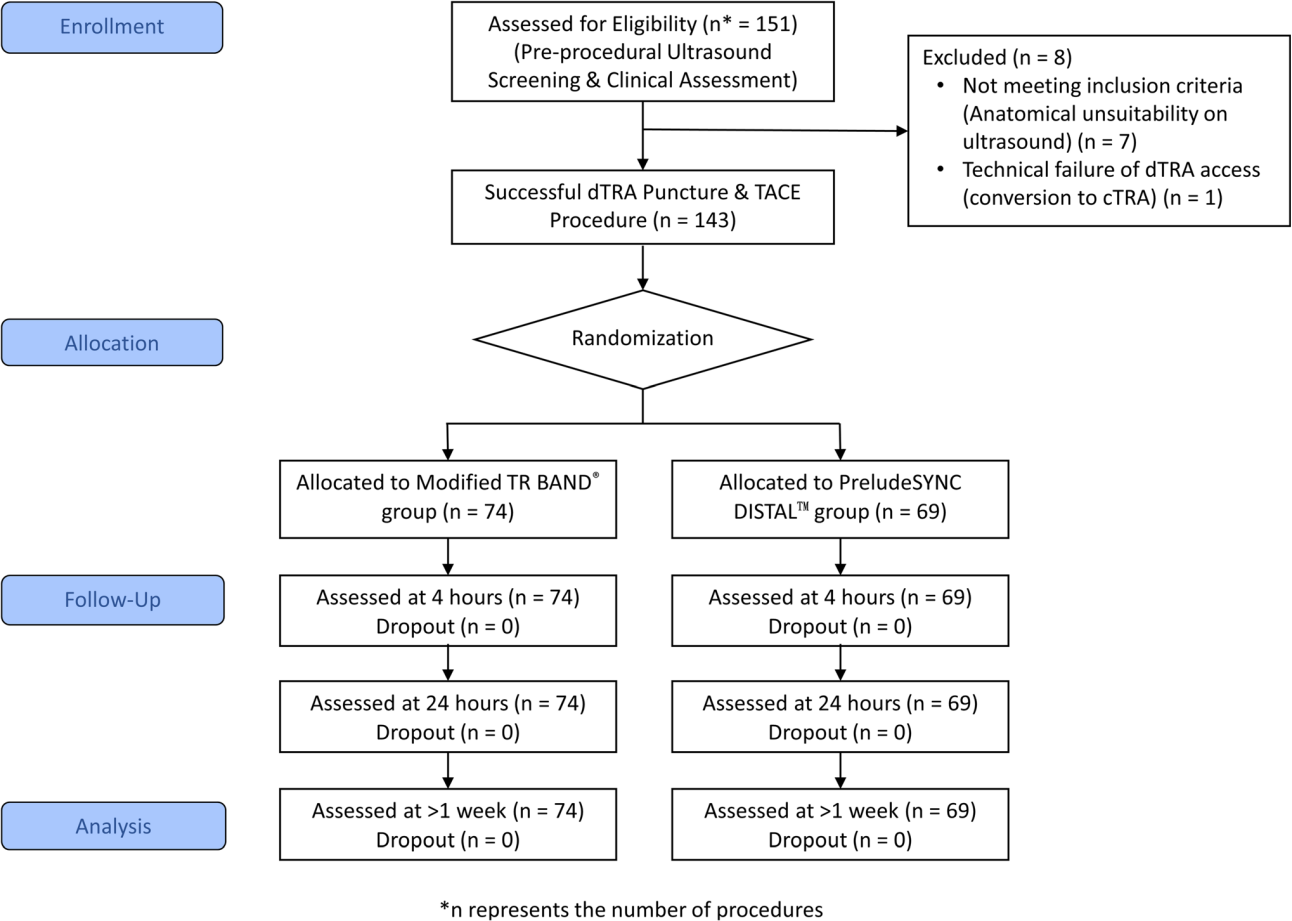
CONSORT flow diagram of Participant enrollment, allocation, and analysis

This prospective, randomized controlled trial is summarized in Fig. 1. Sample size estimation was derived from a prior study reporting RAO rates of 25.0% (19/76) with the TR Band (used at cTRA) and 9.5% (6/63) with the PSD (used at dTRA), with an odds ratio of 3.17 [26]. As this comparison involved different puncture sites, the effect size may overestimate the actual device-related difference. To account for this uncertainty, a power of 90% was selected. Using G*Power for a two-tailed test (α = 0.05, power = 0.90), this calculation yielded a target enrollment of 145 procedures.

Simple randomization was performed at the patient level using computer-generated random numbers, without stratification or blocking. To ensure allocation concealment, group assignment was enclosed in sequentially numbered, opaque, sealed envelopes. After confirming dTRA but before selecting the hemostatic device, the study coordinator opened the next envelope to determine allocation. Patients undergoing subsequent TACE sessions via dTRA maintained their initial randomized assignment throughout the study to prevent crossover effects. Blinding of operators and participants was not feasible due to visible differences between devices. To minimize assessment bias, Doppler ultrasound examinations were conducted according to a standardized protocol with predefined RAO criteria.

### Procedure

Radial access feasibility was assessed using the Allen test to confirm adequate collateral circulation. Patients demonstrating adequate collateral circulation then underwent ultrasound evaluation of the distal radial artery at the anatomical snuffbox to evaluate vessel diameter and course.

All TACE procedures were performed via dTRA (right or left). The access side (left vs. right) was determined by a standardized protocol, prioritizing the patient's non-dominant hand to minimize functional impairment during hemostasis. The dominant hand was used only when pre-procedural ultrasound revealed the non-dominant distal radial artery unsuitable for cannulation. Administration of local anesthesia was performed under ultrasound guidance using a LOGIQ E10 system with a 6–15 MHz linear array transducer (ML6-15; GE HealthCare, Chicago, IL, USA). Puncture was done using an in-plane technique with a 21-G micropuncture set (Merit Medical Systems, South Jordan, UT, USA). Following confirmation of arterial backflow, a 0.018-in. guidewire was advanced and verified in real-time via ultrasound. The needle was then withdrawn, and a 4-Fr radial sheath (Prelude® Mini Access; Merit Medical Systems, South Jordan, UT, USA) was used for all procedures. The sheath entry point was marked using ultrasound-guided orthogonal lines to facilitate accurate placement of the hemostatic device.

A solution of 3,000 U heparin, 2.5 mg verapamil, and 20 mL normal saline was injected through the sheath to prevent vasospasm and thrombosis. This nitroglycerin-free regimen was selected to minimize the risk of procedure-related hypotension. Large-scale randomized trials and meta-analyses in transradial intervention have confirmed that intra-arterial nitroglycerin significantly increases the risk of hypotension [27, 28] without providing a statistically significant reduction in RAO rates compared to placebo [27]. Subsequently, a 4-Fr diagnostic catheter (Performa® Ultimate 1; Merit Medical Systems) and a 2.1-Fr microcatheter (Maestro®; Merit Medical Systems) were advanced, and TACE agents were administered via superselective catheterization. Hemostasis was achieved using either a modified TR Band or PSD, depending on group assignment.

### Intervention

In prior studies, the TR Band was modified for dTRA by removing and repositioning the plastic support plate [20] or simply removing it entirely [29, 30]. In our study, we completely removed the plastic support plate, allowing it to better conform to the anatomical snuffbox and provide uniform compression.

Participants were randomized to receive either the modified TR Band or the PSD. Detailed device characteristics and the standardized application protocol are provided in Appendix A. Demonstration videos are available in Appendices B and C.

A standardized hemostasis protocol was followed: (1) inflation with 15 mL of air (TR Band group) or 10 mL (PSD group), per manufacturer instructions; (2) arterial patency monitoring using the reverse Barbeau test, which confirms adequate compression while preserving arterial flow [31]; and (3) device removal at 4 h postprocedure, followed by Doppler ultrasound assessment. While shorter compression times are feasible in coronary procedures [13], we adopted a conservative 4-h protocol to ensure adequate hemostasis in the cohort of patients with hepatocellular carcinoma, who frequently present with coagulopathy. This duration aligns with the protocol of the recent CONDITION trial, which demonstrated a negligible long-term RAO rate (0.8%) with 4 h of compression [32].

### Data Collection and Outcomes

Radial artery patency was assessed via Doppler ultrasonography at baseline, 4 h (T1), 24 h (T2), and > 1 week (T3). Assessments were performed using color Doppler ultrasonography supplemented by Microvascular Flow Imaging (MVFI) to detect slow flow channels that might be missed by conventional color flow imaging.

The primary endpoint was the incidence of RAO, categorized as: (1) Complete RAO, defined as the total absence of color, spectral, or MVFI signals in the distal radial artery; (2) Partial RAO, defined as the presence of ultrasonographically visible intraluminal thrombus with preserved distal blood flow, or (3) Patent, defined as a patent lumen with no evidence of thrombus or flow compromise.

Secondary endpoints included hemostatic performance and complications such as delayed hemostasis (defined as bleeding requiring compression > 4 h), hematoma, pseudoaneurysm, and neurological symptoms (e.g., paresthesia). Additional secondary endpoints included hemodynamic parameters (peak systolic velocity, pulsatility index, time-averaged mean velocity, and total blood flow) and the feasibility of repeat access, which was evaluated by categorizing procedures based on the patient's cumulative dTRA experience (1st, 2nd, or ≥ 3rd session) on the ipsilateral vessel. Other data collected included demographics, physiological variables, medical history, Charlson Comorbidity Index (CCI), number of puncture attempts, and lifetime TACE sessions.

### Statistical Analysis

We conducted analyses using IBM SPSS Statistics, version 27.0 (IBM Corp., Armonk, NY, USA). Continuous variables were reported as medians (interquartile ranges [IQR]) and compared using the Mann–Whitney *U* test, while categorical variables were analyzed via chi-square test or Fisher's exact test. Longitudinal outcomes (Partial RAO and hemodynamic parameters) were assessed using generalized estimating equations (GEE). Multivariable GEE models were adjusted for baseline characteristics that showed significant differences between groups. Due to limited sample sizes, repeated access data were analyzed descriptively. A *p*-value < 0.05 was considered statistically significant.

## Results

### Patient Characteristics and Procedural Success

Between June 2022 and December 2024, 151 TACE procedures were assessed for eligibility. Eight procedures were excluded (seven due to anatomical unsuitability on ultrasound and one due to technical failure requiring conversion to cTRA), resulting in 143 randomized procedures in 104 patients (Fig. 1). Of these, 74 procedures were assigned to the modified TR Band group and 69 to the PSD group (Table 1). Technical success for TACE via dTRA was achieved in 100% of randomized cases (143/143), with no intra-procedural crossovers to alternative access sites required.

**Table 1:** Baseline characteristics of the study population

Variable	Modified TR Band		PSD		Effect Size ^a^	*p*-value
	*n* (%)	Median (IQR)	*n* (%)	Median (IQR)		
Patient-Level Characteristics	(*n* = 58 patients)		(*n* = 46 patients)			
Sex (male)	44 (75.9%)		36 (78.3%)		− 0.024	0.773
Current smoker (yes)	14 (24.1%)		10 (21.7%)		0.024	0.773
Diabetes (yes)	21 (36.2%)		20 (43.5%)		− 0.073	0.451
Hypertension (yes)	23 (39.7%)		20 (43.5%)		− 0.038	0.694
Hyperlipidemia (yes)	5 (8.6%)		12 (26.1%)		− 0.175	0.017 *
Age (yr)		65.5 (58.8–72.3)		72.0 (63.5–79.0)	0.237	0.016 *
BMI (kg/m^2^)		24.8 (22.9–26.4)		24.9 (23.0–28.6)	0.047	0.628
CCI scores		5.0 (4.0–7.0)		6.0 (5.0–8.0)	0.168	0.087
Lifetime TACE sessions (prior to enrollment)		1.0 (1.0–2.0)		1.0 (1.0–2.0)	0.027	0.781
Procedure-Level Characteristics	(*n* = 74 procedures)		(*n* = 69 procedures)			
Distal radial artery diameter (mm)		1.9 (1.7–2.1)		1.7 (1.5–2.0)	0.251	0.003 *
Vessel-to-sheath ratio		1.4 (1.3–1.6)		1.3 (1.1–1.5)	0.251	0.003 *
Arterial puncture attempts		1.0 (1.0–1.0)		1.0 (1.0–2.0)	0.045	0.592

BMI, body mass index; CCI, Charlson Comorbidity Index; TACE, transarterial chemoembolization. Categorical variables were analyzed using the chi-square test. Continuous variables are presented as Median (IQR) and analyzed using the Mann–Whitney U test due to nonnormal distribution

^a^Effect size is presented as Risk Difference (TR Band—PSD) for categorical variables, and as effect size* r* for continuous variables

*p < 0.05

Baseline characteristics, including sex, smoking status, diabetes, hypertension, body mass index, lifetime TACE sessions, and the number of punctures per procedure, did not significantly differ between the two groups. However, the PSD group had a significantly higher median age (72.0 [IQR 63.5–79.0] vs. 65.5 [IQR 58.8–72.3] years, *p* = 0.016) and a greater prevalence of hyperlipidemia (26.1% vs. 8.6%, *p* = 0.017) than the TR Band group. Additionally, the PSD group presented with a significantly smaller median distal radial artery diameter (1.7 [IQR 1.5–2.0] mm vs. 1.9 [IQR 1.7–2.1] mm,* p* = 0.003), which was reflected in lower vessel-to-sheath ratio (1.3 [IQR 1.1–1.5] vs. 1.4 [IQR 1.3–1.6], *p* = 0.003). The Charlson Comorbidity Index (CCI) scores showed a numerically higher trend in the PSD group (median 6.0 [IQR 5.0–8.0] vs. 5.0 [IQR 4.0–7.0]) but did not reach statistical significance (*p* = 0.087).

### Primary Endpoint: RAO Incidence

**Fig. 2:**
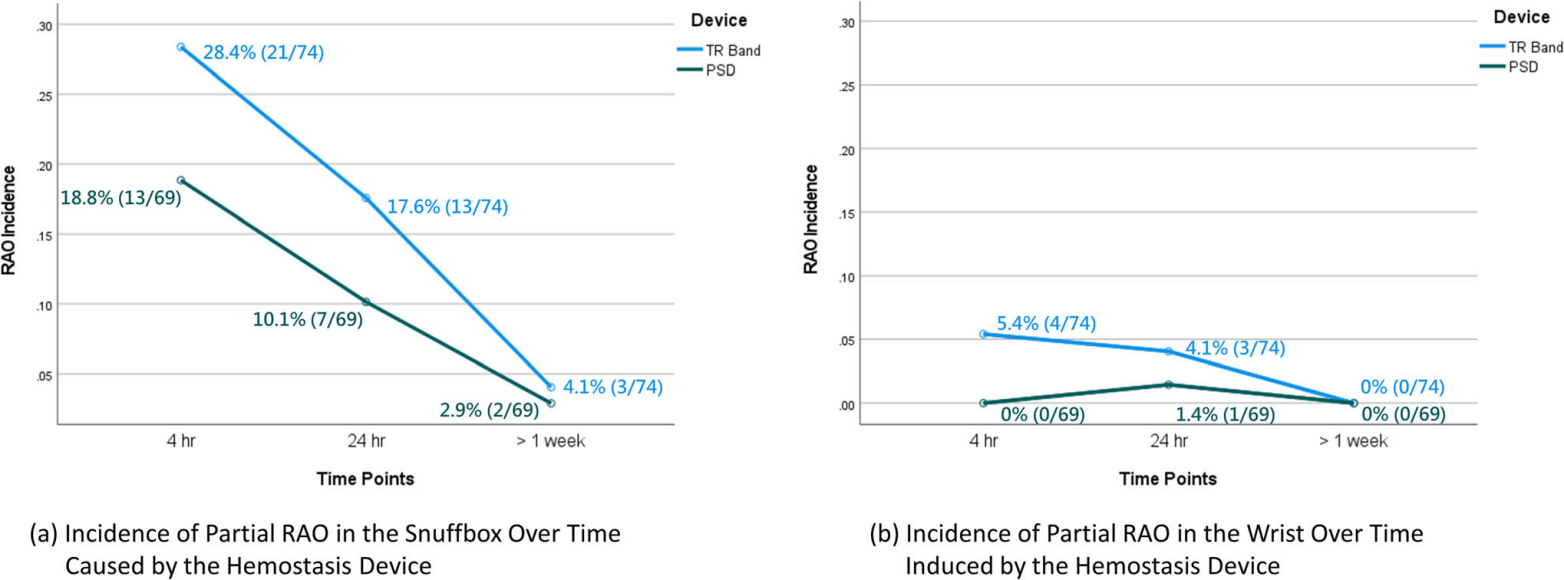
Trends in the incidence of partial Radial artery occlusion (RAO) across three time points (No RAO Observed in the Forearm)

No instances of Complete RAO were observed in either group (0/143). Consequently, the comparative analysis focused exclusively on the incidence of Partial RAO. As illustrated in Fig. 2, the distal radial artery (snuffbox) was the predominant site of Partial RAO, with incidence peaking at 4 h after procedure (n = 34) and declining thereafter. Specifically, 29 of the 34 cases (85.3%) resolved spontaneously by the final follow-up (> 1 week). The TR Band group consistently showed numerically higher Partial RAO rates across all time points (28.4% vs. 18.8% at 4 h, 17.6% vs. 10.1% at 24 h, and 4.1% vs. 2.9% beyond 1 week), although these differences were not statistically significant. No occlusion was detected in the proximal radial artery (wrist). All observed Partial RAO cases were asymptomatic and clinically silent. No patient experienced signs of hand ischemia, resting pain, tissue necrosis, or loss of hand function. Thus, all ultrasound-detected occlusions were classified as subclinical minor complications.

GEE analysis demonstrated no significant difference in overall Partial RAO incidence between the two hemostatic devices, even after adjusting for age, hyperlipidemia, CCI, and vessel-to-sheath ratio. Furthermore, interaction terms between device type and time (device × T2: *p* = 0.804; device × T3: *p* = 0.840) were not statistically significant, indicating that temporal trends in Partial RAO incidence were similar between the two groups (Table 2).

**Table 2:** Generalized estimating equation analysis of factors associated with partial RAO at the distal radial artery

Variable	*Adjusted Model*^a^			*Unadjusted Model*		
	Coefficient^a^	Standard Error^a^	*p*-value^a^	Coefficient	Standard Error	*p*-value
Hemostasis device (PSD vs. TR Band)	− 0.677	0.392	0.084	− 0.535	0.402	0.183
Time point (T3 vs. T1)	− 2.257	0.573	< 0.001	− 2.238	0.561	< 0.001
Time point (T2 vs. T1)	− 0.633	0.293	0.030	− 0.620	0.286	0.030
Hyperlipidemia	− 0.440	0.533	0.410			
Age (yr)	0.006	0.018	0.749			
CCI scores	0.104	0.112	0.355			
Vessel-to-sheath ratio	− 0.753	0.790	0.340			
Hemostasis device × T3 interaction	0.178	0.944	0.850	0.187	0.929	0.840
Hemostasis device × T2 interaction	− 0.100	0.411	0.808	− 0.101	0.405	0.804

CCI, Charlson Comorbidity Index; T1, 4 h postprocedure; T2, 24 h postprocedure; T3, > 1 week postprocedure. Covariates were selected a priori based on clinical relevance and baseline imbalances. Inclusion of these variables did not materially change the effect of the hemostasis device

^a^Adjusted estimates for the four significant independent variables

### Secondary Endpoints: Hemostatic Performance and Complications

Hemostatic outcomes and access-site complications are summarized in Table 3. Overall, the incidence of complications was low in both groups. There were no statistically significant differences in the rates of hematoma (14.9% vs. 18.8%, *p* = 0.525), pseudoaneurysm (1.4% vs. 1.4%, *p* = 1.000), or paresthesia (4.1% vs. 2.9%, *p* = 1.000). Regarding hemostatic efficiency, the TR Band group exhibited a numerically higher rate of delayed hemostasis (defined as bleeding requiring compression for more than 4 h) compared with the PSD group (5.4% vs. 0.0%); however, this difference was not statistically significant (*p* = 0.121).

**Table 3:** Safety endpoints and clinical complications

Endpoint	Modified TR Band (n = 74)	PSD (n = 69)	Risk Difference (95% CI)	*p*-value
Hemostasis-Related				
Delayed Hemostasis (> 4 h)^1^	4 (5.4%)	0 (0.0%)	5.4% (0.3%–10.6%)	0.121^2^
Access-Site Complications				
Hematoma	11 (14.9%)	13 (18.8%)	− 4.0% (− 16.3%–8.3%)	0.525
Pseudoaneurysm	1 (1.4%)	1 (1.4%)	− 0.1% (− 4.0%–3.8%)	1.000^2^
Neurological Complications				
Paresthesia (Numbness)	3 (4.1%)	2 (2.9%)	1.2% (− 4.8%–7.1%)	1.000^2^
Thumb Swelling/Weakness	0 (0.0%)	1 (1.4%)	− 1.4% (− 4.3%–1.4%)	0.483 ^2^

^1^Includes 4 cases (5 h, 6 h, 6 h, 7 h) in the TR Band group. ^2^*p*-value calculated using Fisher's exact test

### Hemodynamics and Repeat Access Feasibility

Time-series data on hemodynamic parameters are presented in Appendix D. According to color Doppler ultrasound assessments, both groups followed similar postprocedure recovery trajectories. A correlation between hemodynamic fluctuations and clinical outcomes could not be established, as there were no instances of symptomatic ischemia or hand dysfunction (0/143). This absence of association confirms that the observed hemodynamic variations remained strictly subclinical.

Despite these overall trends, at the late postprocedure stage (> 1 week), the PSD group exhibited significantly higher forearm peak systolic velocity than the TR Band group (Appendix E). This finding was supported by a significant device × time interaction in the GEE model (PSD × T3: *p* = 0.031). No such interaction was observed for other hemodynamic parameters, and overall trends remained consistent across time points. After adjusting for age, hyperlipidemia, CCI, and the vessel-to-sheath ratio, results were unchanged.

To assess the impact of repeated cannulation, procedures were categorized by the patient's cumulative dTRA experience: 1st dTRA procedure (n = 88), 2nd dTRA procedure (n = 34), and ≥ 3rd dTRA procedure (n = 21). At 4 h after procedure, the incidence of Partial RAO was 26.1% in the 1st procedure group, 23.5% in the 2nd procedure group, and 14.3% in the ≥ 3rd procedure group. Although a decreasing trend was observed, no formal statistical inference was performed due to the limited sample size in the repeat access subgroups.

## Discussion

In this prospective, randomized trial comparing the modified TR Band and PSD for TACE, we observed no significant differences in Partial RAO incidence or vascular patency at any time point.

Our results contrast with earlier reports suggesting higher occlusion risks with the TR Band. These discrepancies likely stem from methodological heterogeneity. The prior comparative study was retrospective and included both conventional and distal access [26], whereas our trial was prospective and focused explicitly on dTRA. Notably, our observed low incidence of RAO aligns closely with emerging evidence. The recent large-scale CONDITION trial reported a long-term RAO rate of only 0.8% for dTRA [32], and a recent non-coronary interventional radiology cohort similarly reported a 0% occlusion rate [16]. Collectively, these data suggest that early retrospective studies may have overestimated the risks of RAO. Modern dTRA techniques, including micropuncture sets and slender sheaths, likely contribute to the favorable patency outcomes observed in recent trials.

Doppler ultrasonography, which offers higher sensitivity compared with pulse palpation, was used to detect Partial RAO, defined as the presence of thrombus with preserved flow. Both groups experienced transient reductions in flow that resolved over time, consistent with a prior meta-analysis reporting a decline in RAO rates from 7.7% at 24 h to 5.5% at 1 week [24]. All Partial RAO cases were subclinical findings, and 85.3% (29/34) resolved spontaneously without any intervention. At the final follow-up (> 1 week), the Partial RAO rate at the distal radial artery was 3.5% (5/143), with no occlusions observed at the wrist or forearm. These findings align with previous reports suggesting that distal radial access minimizes trauma to the proximal radial artery, thereby reducing the risk of injury to the trunk.

Regarding clinical complications, no meaningful adverse events were observed. Across 143 procedures, there were no cases (0%) of radial artery occlusion, hand ischemia, tissue necrosis, or permanent nerve injury. As detailed in Table 3, minor complications such as hematoma and transient paresthesia were rare and did not differ significantly between groups. This suggests that even when Partial RAO occurs, the preservation of the superficial palmar arch via dTRA prevents ischemic sequelae.

Beyond device-related factors, anatomical and procedural variables may also influence patency outcomes. A smaller vessel-to-sheath ratio has been associated with increased intimal injury and a higher risk of RAO in earlier studies [24]. In this trial, the TR Band group had a higher median vessel-to-sheath ratio compared with the PSD group. While smaller ratios have been linked to an increased incidence of RAO [15], our adjusted analyses did not reveal a statistically significant association. Regarding hemodynamic parameters, we observed a statistically significant interaction between device and time for forearm peak systolic velocity (*p* = 0.031, Appendix E). However, we interpret this finding with caution and consider it likely a chance observation (Type I error) resulting from multiple statistical comparisons rather than an actual device effect. This variation was an isolated finding, not observed in other hemodynamic parameters or at other time points. Given the absence of corresponding differences in RAO incidence or clinical ischemic symptoms, this statistical anomaly does not appear to carry clinical significance. Other patient-related risk factors, including advanced age, female sex, low body mass index, diabetes, and dyslipidemia, have also been implicated in RAO risk [15, 33, 34]. Although our study adjusted for age and hyperlipidemia, the limited event rate precluded comprehensive risk factor analysis.

Despite these limitations in risk factor analysis, this study contributes to an evolving evidence base. Currently, evidence characterizing the safety and feasibility of dTRA for TACE is limited to a few case reports [19] and retrospective studies [17, 35, 36], underscoring the need for prospective trials. This study addresses that gap by providing clinical insights into the incidence trends of Partial RAO and the feasibility of repeated radial artery use for TACE.

Given similar RAO outcomes, device selection depends on practical factors. The modified TR Band is cost-efficient and versatile for both hands and access sites, with well-documented feasibility [20]. In contrast, although the PSD requires stocking separate units for the left and right hands, it features an anatomically contoured frame that provides superior stability [21]. Therefore, selection should weigh operator preference, anatomy, budget constraints, and inventory demands. Depending on these non-clinical considerations, either device may be suitable, although the TR Band offers greater flexibility.

Our findings have several limitations. First, the study is underpowered due to unexpectedly low event rates (0% Complete RAO); thus, negative results indicate a failure to detect differences rather than equivalence. Second, the single-center, HCC-specific design limits generalizability. Third, despite statistical adjustments for baseline imbalances (age, hyperlipidemia, vessel-to-sheath ratio), residual confounding cannot be excluded. Fourth, exclusive 4-Fr use limits extrapolation to larger devices. Fifth, small sample sizes for repeat dTRA (*n* = 21 for ≥ 3 sessions) necessitated descriptive analysis, precluding definitive conclusions regarding sustained patency. Finally, while complications were recorded, we did not systematically assess pain scores and patient satisfaction.

## Conclusion

This randomized trial demonstrated similar safety profiles and patency outcomes for the modified TR Band and PSD in TACE, with no significant differences observed. Device selection should be based on both clinical outcomes and practical factors, including cost, availability, and institutional resources. Larger multicenter studies are warranted to validate these findings.

## Supplementary Information

Below is the link to the electronic supplementary material.

Supplementary file1 (DOCX 660 kb)

Supplementary file2 (MP4 62107 kb)

Supplementary file3 (MP4 73592 kb)

## Data Availability

The dataset supporting the results of this article is included within the article.
