# Supplementary material for: Comparing Two Distal Radial Hemostatic Devices for Radial Artery Patency Post-TACE: A Randomized Trial
Source: Cardiovasc Intervent Radiol. 2026 Jan 23;49(5):896–905. doi: 10.1007/s00270-025-04337-8 (PMC13156157; doi:10.1007/s00270-025-04337-8)
Supplement: Supplementary file 1 — Supplementary file1 (DOCX 660 kb) [file 270_2025_4337_MOESM1_ESM.docx]

**
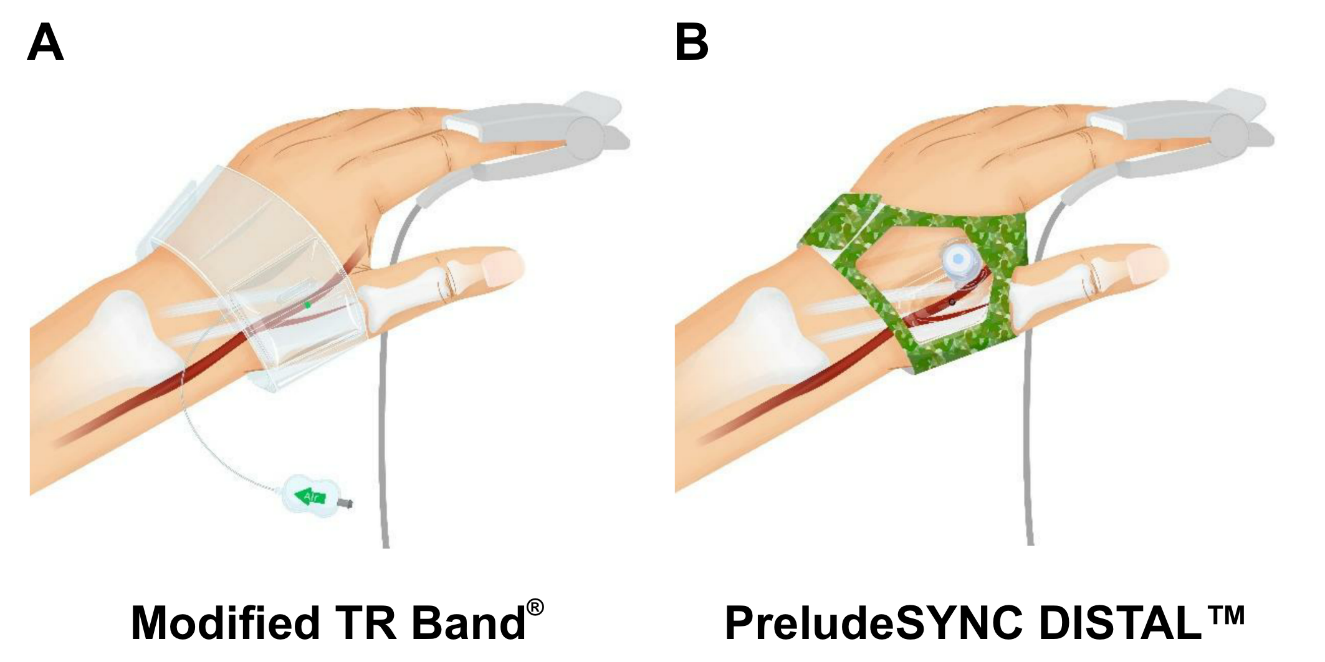
Appendix A.** Device Characteristics and Hemostasis Protocol

1. Device Characteristics

(A) Modified TR Band^®^: The TR Band, originally designed for conventional radial access, was modified for distal transradial access (dTRA) by completely removing the plastic support plate. This modification eliminates the rigid structure, thereby improving conformity to the anatomical snuffbox and ensuring uniform compression over the concave surface. The device utilizes an inflatable balloon system with an initial inflation of 15 mL of air.

(B) PreludeSYNC DISTAL™ (PSD): The PSD is a dedicated device specifically designed for hemostasis at the anatomical snuffbox. It features a specialized compression balloon optimized for the dorsal hand anatomy and a transparent window allowing direct visualization of the puncture site. The nominal air inflation volume is 10 mL.

2. Hemostasis Protocol

A standardized hemostasis protocol was applied to both groups following the TACE procedure:

1. Site Localization: Under ultrasound guidance, a cross was marked on the skin to precisely indicate the vascular subcutaneous entry site.
2. Device Application:
   - Modified TR Band Group: The device was positioned such that the green marker of the balloon aligned with the skin marking. As noted above, the support plate was removed to optimize fit. An initial volume of 15 mL of air was injected into the balloon simultaneously with sheath removal to provide adequate initial compression.
   - PSD Group: The device was applied according to the manufacturer’s instructions, adjusting the center of the balloon to match the skin marking. An initial volume of 10 mL of air was injected simultaneously with sheath removal.
3. Patent Hemostasis Assessment (Reverse Barbeau Test): A pulse oximeter was placed on the index finger. The ulnar artery was manually compressed to assess the patency of the distal radial artery.
   - If the pulse oximetry waveform was extremely weak or disappeared (indicating occlusion), 1 mL of air was released from the balloon.
   - This titration process continued until an acceptable oxygenation level or waveform (Barbeau waveform types A-C) was achieved while maintaining hemostasis.
   - If hemorrhage occurred after air release, air was re-injected to re-establish hemostasis.
4. Follow-up: The device was removed 4 hours after procedure, and radial artery patency was confirmed via Doppler ultrasonography.

**Appendix B.** Video Demonstration of the Application Procedure for the Modified TR Band^®^

This video illustrates the placement and initial inflation of the modified TR Band^®^ to ensure a consistent and standardized method for securing hemostasis.

**Appendix C.** Video Demonstration of the Application Procedure for PreludeSYNC DISTAL™ (PSD)

This video illustrates the placement and initial inflation of PreludeSYNC DISTAL™ (PSD) to ensure a consistent and standardized method for securing hemostasis.

**Appendix D.** Trends in Hemodynamic Parameters Across Time According to Measurement Site and Hemostasis Device

| 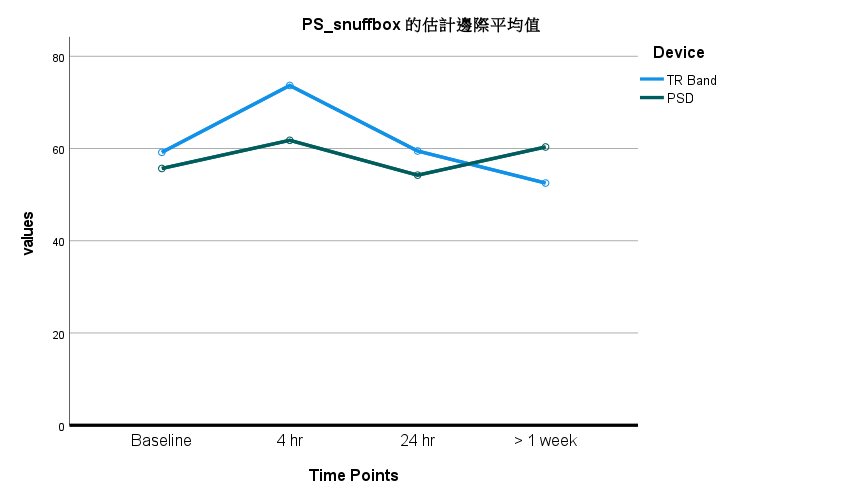(a) Peak Systolic Velocity in the Snuffbox Over Time | 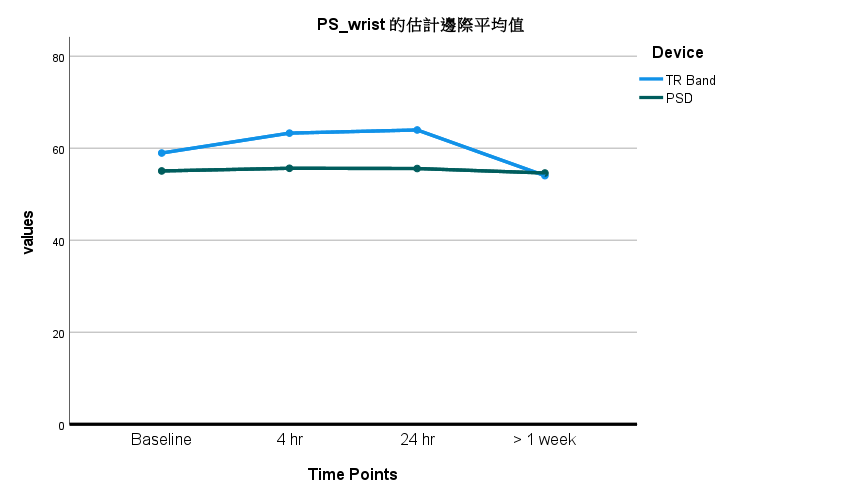 (b) Peak Systolic Velocity in the Wrist Over Time | 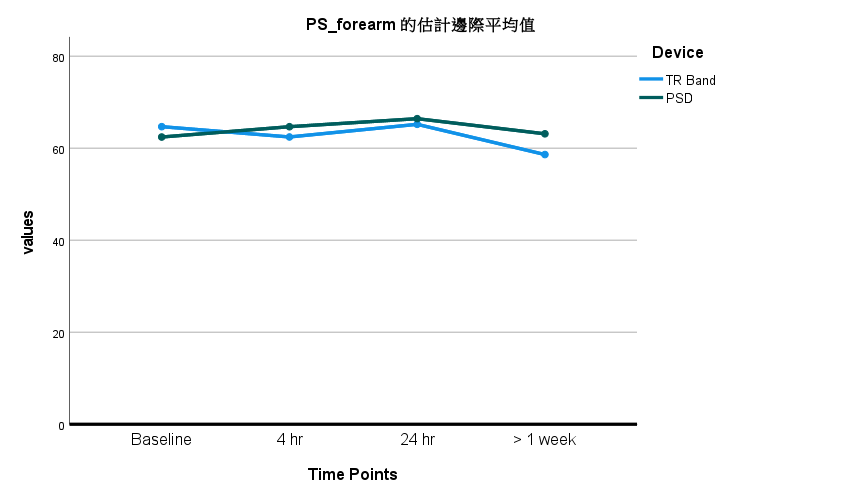(c) Peak Systolic Velocity in the Forearm Over Time |
| --- | --- | --- |
| 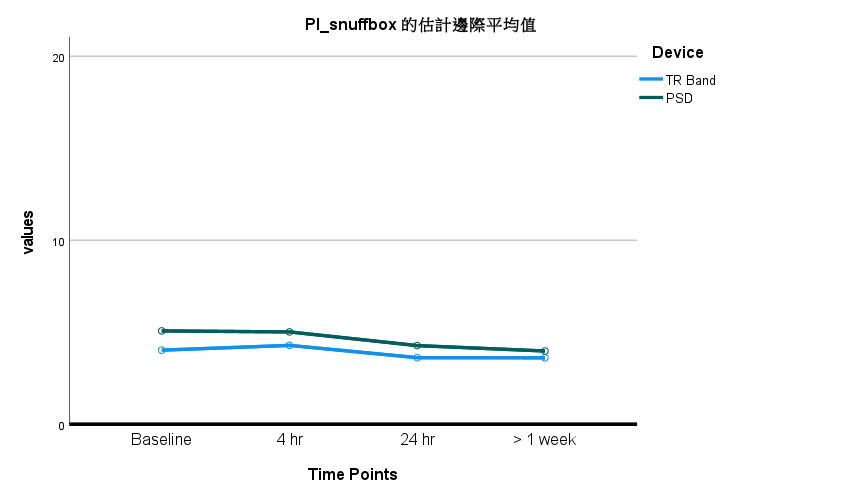(d) Pulsatility Index in the Snuffbox Over Time | 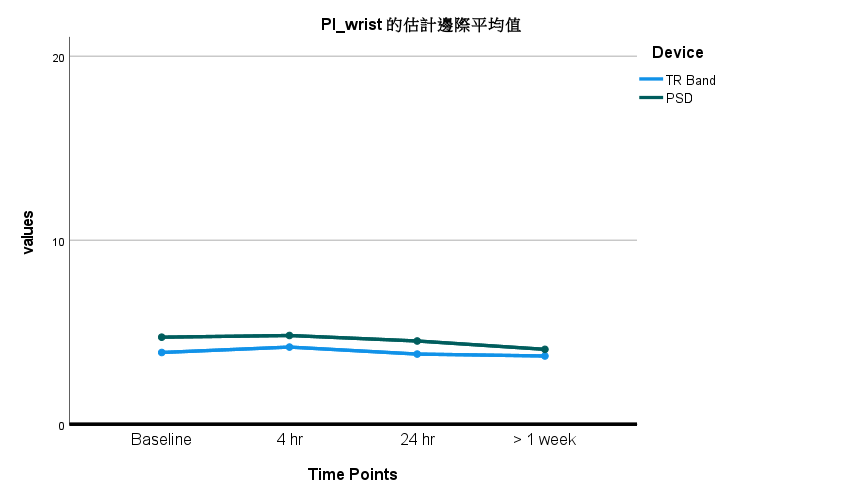(e) Pulsatility Index in the Wrist Over Time | 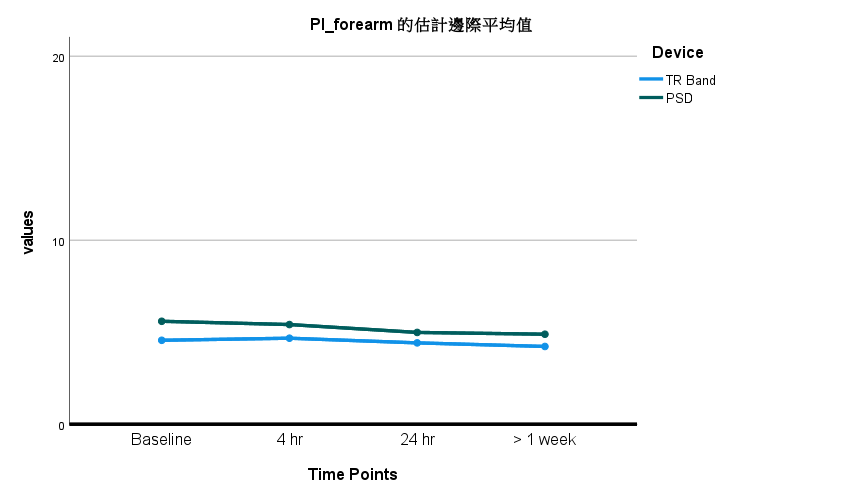(f) Pulsatility Index in the Forearm Over Time |
| 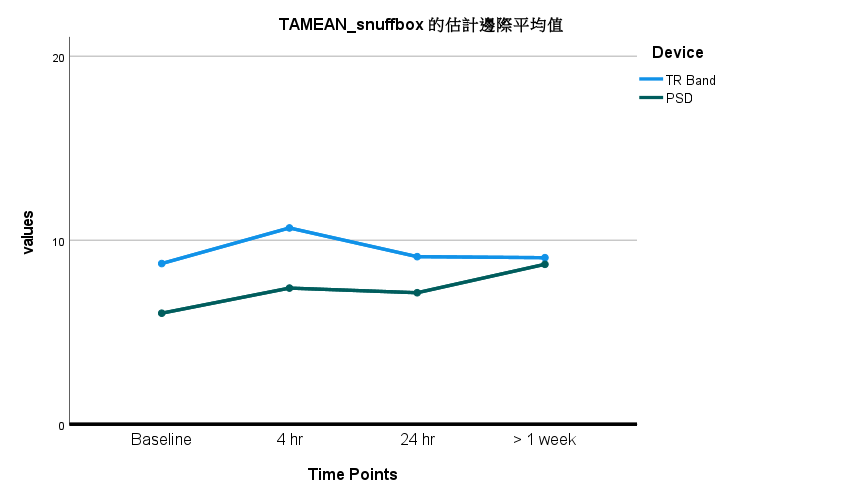(g) Time-Averaged Mean Velocity in the Snuffbox Over Time | 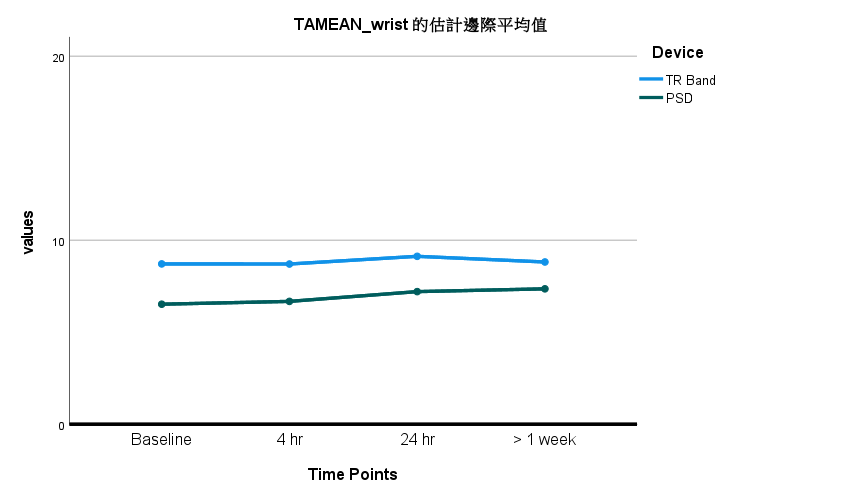(h) Time-Averaged Mean Velocity in the Wrist Over Time | 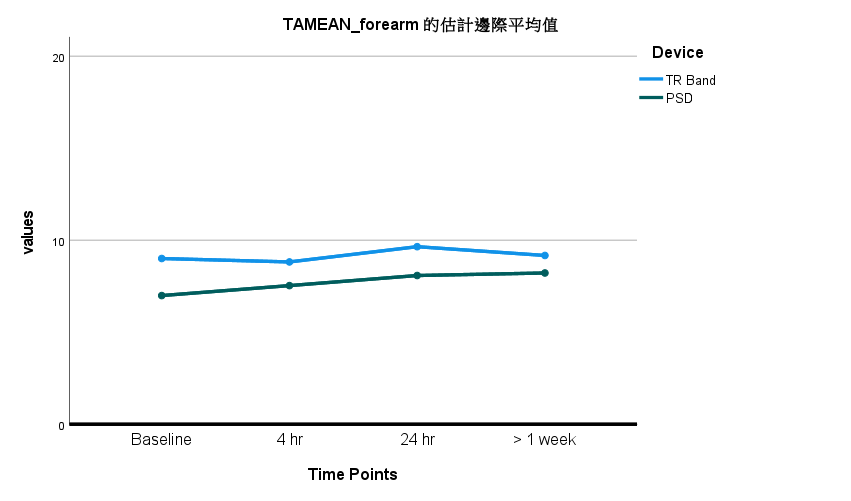(i) Time-Averaged Mean Velocity in the Forearm Over Time |
| 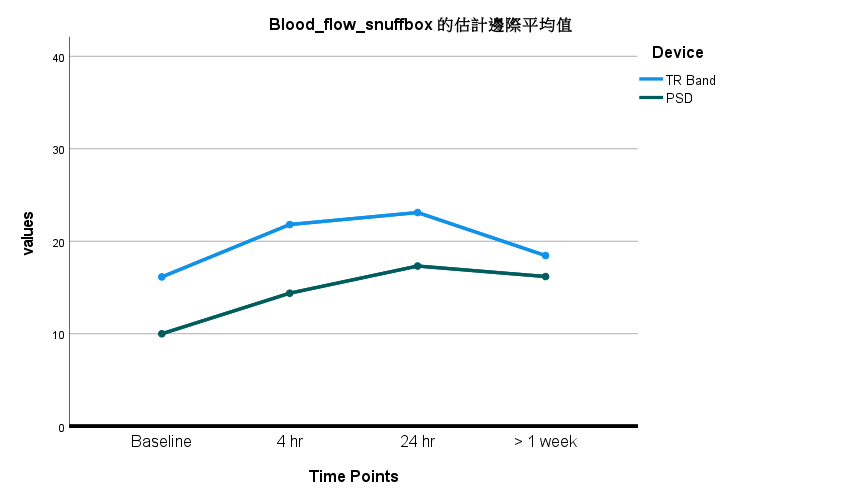(j) Blood Flow in the Snuffbox Over Time | 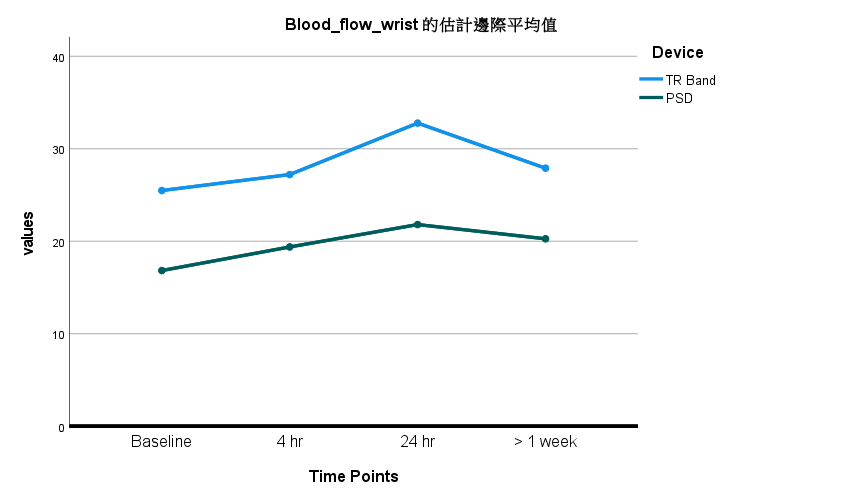(k) Blood Flow in the Wrist Over Time | 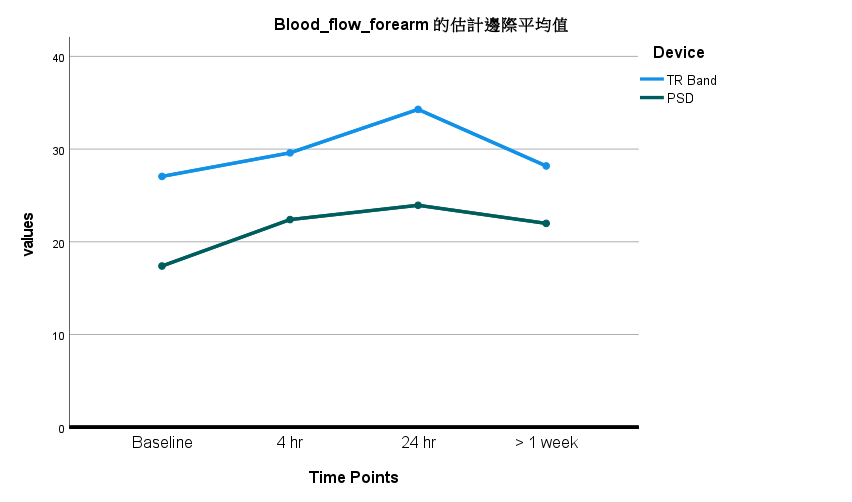(l) Blood Flow in the Forearm Over Time |

**Appendix E**. Generalized Estimating Equation Analysis of the Effects of the Hemostasis Device on Hemodynamic Parameters at Different Measurement Sites

| Measurement site | Variables | Adjusted Model ^a^ | | | Unadjusted Model | | |
| --- | --- | --- | --- | --- | --- | --- | --- |
|  |  | Coefficient^a^ | Standard Error^a^ | p-value^a^ | Coefficient | Standard Error | p-value |
| Peak systolic velocity (PSV) |  |  |  |  |  |  |  |
| Snuffbox | Hemostasis device (PSD vs. TR Band) | −3.952 | 3.9765 | 0.320 | −3.379 | 3.8676 | 0.382 |
|  | Time point (T3 vs. T0) | −6.340 | 4.2711 | 0.138 | −6.364 | 4.2999 | 0.139 |
|  | Time point (T2 vs. T0) | −0.224 | 3.9806 | 0.955 | −0.053 | 3.9925 | 0.989 |
|  | Time point (T1 vs. T0) | 14.138 | 5.0699 | 0.005 | 14.336 | 5.0662 | 0.005 |
|  | PSD × T3 interaction | 11.052 | 6.2773 | 0.078 | 11.138 | 6.2917 | 0.077 |
|  | PSD × T2 interaction | −1.210 | 5.2807 | 0.819 | −1.496 | 5.2727 | 0.777 |
|  | PSD × T1 interaction | −8.043 | 6.8624 | 0.241 | −8.284 | 6.8568 | 0.227 |
| Wrist | Hemostasis device (PSD vs. TR Band) | −3.654 | 3.0486 | 0.231 | −4.001 | 3.0149 | 0.185 |
|  | Time point (T3 vs. T0) | −5.496 | 3.0600 | 0.072 | −5.394 | 3.0730 | 0.079 |
|  | Time point (T2 vs. T0) | 4.859 | 3.1901 | 0.128 | 4.905 | 3.1847 | 0.124 |
|  | Time point (T1 vs. T0) | 4.144 | 3.5813 | 0.247 | 4.213 | 3.5929 | 0.241 |
|  | PSD × T3 interaction | 4.906 | 3.9600 | 0.215 | 4.766 | 3.9712 | 0.230 |
|  | PSD × T2 interaction | −4.175 | 3.8436 | 0.277 | −4.253 | 3.8438 | 0.269 |
|  | PSD × T1 interaction | −3.611 | 4.5166 | 0.424 | −3.693 | 4.5281 | 0.415 |
| Forearm | Hemostasis device (PSD vs. TR Band) | −3.905 | 2.7698 | 0.159 | −2.183 | 2.8195 | 0.439 |
|  | Time point (T3 vs. T0) | −6.331 | 2.5936 | 0.015 | −6.416 | 2.6023 | 0.014 |
|  | Time point (T2 vs. T0) | 0.241 | 2.3209 | 0.917 | 0.304 | 2.3132 | 0.896 |
|  | Time point (T1 vs. T0) | −2.804 | 2.4145 | 0.245 | −2.775 | 2.4280 | 0.253 |
|  | PSD × T3 interaction | 7.223 | 3.4576 | 0.037 | 7.477 | 3.4636 | 0.031 |
|  | PSD × T2 interaction | 3.356 | 3.3294 | 0.314 | 3.375 | 3.3248 | 0.310 |
|  | PSD × T1 interaction | 4.701 | 3.5127 | 0.181 | 4.742 | 3.5293 | 0.179 |
| Pulsatility index (PI) |  |  |  |  |  |  |  |
| Snuffbox | Hemostasis device (PSD vs. TR Band) | 0.723 | 0.2980 | 0.015 | 1.063 | 0.3078 | 0.001 |
|  | Time point (T3 vs. T0) | −0.393 | 0.3325 | 0.237 | −0.420 | 0.3349 | 0.210 |
|  | Time point (T2 vs. T0) | −0.428 | 0.2353 | 0.069 | −0.417 | 0.2349 | 0.076 |
|  | Time point (T1 vs. T0) | 0.283 | 0.3066 | 0.356 | 0.296 | 0.3067 | 0.334 |
|  | PSD × T3 interaction | −0.713 | 0.4292 | 0.096 | −0.671 | 0.4326 | 0.121 |
|  | PSD × T2 interaction | −0.376 | 0.3294 | 0.254 | −0.377 | 0.3289 | 0.252 |
|  | PSD × T1 interaction | −0.352 | 0.4027 | 0.381 | −0.361 | 0.4032 | 0.370 |
| Wrist | Hemostasis device (PSD vs. TR Band) | 0.544 | 0.2565 | 0.034 | 0.837 | 0.2433 | 0.001 |
|  | Time point (T3 vs. T0) | −0.165 | 0.2629 | 0.531 | −0.171 | 0.2636 | 0.517 |
|  | Time point (T2 vs. T0) | −0.090 | 0.2037 | 0.658 | −0.081 | 0.2027 | 0.691 |
|  | Time point (T1 vs. T0) | 0.301 | 0.2483 | 0.225 | 0.307 | 0.2469 | 0.214 |
|  | PSD × T3 interaction | −0.460 | 0.3524 | 0.192 | −0.434 | 0.3552 | 0.222 |
|  | PSD × T2 interaction | −0.142 | 0.3956 | 0.720 | −0.147 | 0.3946 | 0.710 |
|  | PSD × T1 interaction | −0.206 | 0.3430 | 0.549 | −0.217 | 0.3421 | 0.527 |
| Forearm | Hemostasis device (PSD vs. TR Band) | 0.583 | 0.2845 | 0.040 | 1.034 | 0.2939 | 0.000 |
|  | Time point (T3 vs. T0) | −0.304 | 0.2867 | 0.289 | −0.331 | 0.2881 | 0.250 |
|  | Time point (T2 vs. T0) | −0.145 | 0.2318 | 0.531 | −0.135 | 0.2304 | 0.558 |
|  | Time point (T1 vs. T0) | 0.151 | 0.2530 | 0.551 | 0.156 | 0.2511 | 0.534 |
|  | PSD × T3 interaction | −0.418 | 0.4181 | 0.318 | −0.370 | 0.4235 | 0.383 |
|  | PSD × T2 interaction | −0.520 | 0.3279 | 0.113 | −0.515 | 0.3280 | 0.117 |
|  | PSD × T1 interaction | −0.329 | 0.3668 | 0.369 | −0.329 | 0.3656 | 0.368 |
| Time-averaged mean velocity (TAMEAN) | |  |  |  |  |  |  |
| Snuffbox | Hemostasis device (PSD vs. TR Band) | −1.988 | 0.7663 | 0.009 | −2.697 | 0.7473 | <0.001 |
|  | Time point (T3 vs. T0) | 0.311 | 1.1451 | 0.786 | 0.370 | 1.1552 | 0.749 |
|  | Time point (T2 vs. T0) | 0.337 | 0.9520 | 0.723 | 0.324 | 0.9487 | 0.732 |
|  | Time point (T1 vs. T0) | 1.857 | 1.1463 | 0.105 | 1.855 | 1.1475 | 0.106 |
|  | PSD × T3 interaction | 2.379 | 1.4553 | 0.102 | 2.298 | 1.4610 | 0.116 |
|  | PSD × T2 interaction | 0.801 | 1.0986 | 0.466 | 0.771 | 1.0935 | 0.480 |
|  | PSD × T1 interaction | −0.466 | 1.3631 | 0.733 | −0.487 | 1.3646 | 0.721 |
| Wrist | Hemostasis device (PSD vs. TR Band) | −1.567 | 0.6149 | 0.011 | −2.213 | 0.6129 | <0.001 |
|  | Time point (T3 vs. T0) | −0.007 | 0.8983 | 0.994 | 0.030 | 0.9033 | 0.973 |
|  | Time point (T2 vs. T0) | 0.396 | 0.7370 | 0.591 | 0.389 | 0.7356 | 0.597 |
|  | Time point (T1 vs. T0) | −0.059 | 0.6515 | 0.928 | −0.057 | 0.6513 | 0.930 |
|  | PSD × T3 interaction | 0.864 | 1.0244 | 0.399 | 0.799 | 1.0304 | 0.438 |
|  | PSD × T2 interaction | 0.352 | 0.8522 | 0.680 | 0.342 | 0.8509 | 0.688 |
|  | PSD × T1 interaction | 0.200 | 0.8061 | 0.804 | 0.204 | 0.8061 | 0.800 |
| Forearm | Hemostasis device (PSD vs. TR Band) | −1.355 | 0.6314 | 0.032 | −2.021 | 0.6470 | 0.002 |
|  | Time point (T3 vs. T0) | 0.027 | 0.8358 | 0.974 | 0.065 | 0.8375 | 0.939 |
|  | Time point (T2 vs. T0) | 0.605 | 0.7252 | 0.404 | 0.595 | 0.7232 | 0.410 |
|  | Time point (T1 vs. T0) | −0.298 | 0.6060 | 0.623 | −0.294 | 0.6060 | 0.628 |
|  | PSD × T3 interaction | 1.252 | 1.0152 | 0.218 | 1.196 | 1.0185 | 0.240 |
|  | PSD × T2 interaction | 0.535 | 0.8845 | 0.546 | 0.519 | 0.8837 | 0.557 |
|  | PSD × T1 interaction | 0.798 | 0.7345 | 0.277 | 0.796 | 0.7349 | 0.279 |
| Blood flow |  |  |  |  |  |  |  |
| snuffbox | Hemostasis device (PSD vs. TR Band) | −3.292 | 1.5491 | 0.034 | −6.058 | 1.6959 | <0.001 |
|  | Time point (T3 vs. T0) | 2.142 | 2.5703 | 0.405 | 2.519 | 2.6215 | 0.337 |
|  | Time point (T2 vs. T0) | 6.314 | 2.2787 | 0.006 | 6.489 | 2.2751 | 0.004 |
|  | Time point (T1 vs. T0) | 5.530 | 2.4824 | 0.026 | 5.473 | 2.4917 | 0.028 |
|  | PSD × T3 interaction | 4.049 | 3.1343 | 0.196 | 3.588 | 3.1546 | 0.255 |
|  | PSD × T2 interaction | 0.974 | 2.8278 | 0.731 | 0.568 | 2.8191 | 0.840 |
|  | PSD × T1 interaction | −1.057 | 2.9234 | 0.718 | −1.124 | 2.9358 | 0.702 |
| Wrist | Hemostasis device (PSD vs. TR Band) | −6.153 | 2.5648 | 0.016 | −8.692 | 2.6662 | 0.001 |
|  | Time point (T3 vs. T0) | 1.979 | 4.0004 | 0.621 | 2.145 | 4.0105 | 0.593 |
|  | Time point (T2 vs. T0) | 7.032 | 2.9747 | 0.018 | 7.154 | 2.9940 | 0.017 |
|  | Time point (T1 vs. T0) | 1.565 | 2.6293 | 0.552 | 1.616 | 2.6380 | 0.540 |
|  | PSD × T3 interaction | 1.284 | 4.5296 | 0.777 | 1.057 | 4.5345 | 0.816 |
|  | PSD × T2 interaction | −1.986 | 3.3290 | 0.551 | −2.283 | 3.3514 | 0.496 |
|  | PSD × T1 interaction | 0.974 | 3.1516 | 0.757 | 0.919 | 3.1582 | 0.771 |
| Forearm | Hemostasis device (PSD vs. TR Band) | −6.652 | 2.7646 | 0.016 | −9.665 | 3.0208 | 0.001 |
|  | Time point (T3 vs. T0) | 0.848 | 3.8538 | 0.826 | 0.989 | 3.8507 | 0.797 |
|  | Time point (T2 vs. T0) | 7.041 | 3.1083 | 0.024 | 7.215 | 3.1289 | 0.021 |
|  | Time point (T1 vs. T0) | 1.907 | 2.8567 | 0.504 | 1.924 | 2.8597 | 0.501 |
|  | PSD × T3 interaction | 3.565 | 4.5336 | 0.432 | 3.381 | 4.5299 | 0.455 |
|  | PSD × T2 interaction | −0.385 | 3.6167 | 0.915 | −0.685 | 3.6361 | 0.850 |
|  | PSD × T1 interaction | 2.946 | 3.3812 | 0.384 | 2.934 | 3.3844 | 0.386 |

^a^ Indicates adjusted estimates for four significant independent variables.

T0: baseline (pre-procedure), T1: 4 hours postprocedure, T2: 24 hours postprocedure, T3: > 1 week postprocedure.
